# Pediatric vs. Adult Prodrome and Postdrome: A Window on Migraine Pathophysiology?

**DOI:** 10.3389/fneur.2019.00199

**Published:** 2019-03-08

**Authors:** Jean-Christophe Cuvellier

**Affiliations:** Division of Pediatric Neurology, Department of Pediatrics, Lille Faculty of Medicine and Children's Hospital, Lille, France

**Keywords:** migraine, prodrome, postdrome, child, adolescent, adult, pathophysiology

## Abstract

Few studies have been conducted on the prodromal and postdromal phases of the migraine attack in children and adolescents. Using a questionnaire, we found that 67% of 103 children and adolescents with migraine reported at least one prodromal symptom, with a mean number per subject of 1.8 (median 2.2). The most frequently reported prodromal symptoms were face changes, fatigue and irritability. In pediatric patients selected as having prodrome, fatigue, mood change and neck stiffness were the most frequently reported prodromal symptoms. Using a different design, Laurell et al. found that 71% of 137 pediatric patients reported at least one prodromal symptom with a mean number per subject of 1.9 ± 2.0. Studying postdrome was fraught with unexpected difficulties as our preliminary research showed. Patients reported 2 groups of symptoms occurring during the resolution phase of the headache: symptoms whose onset was *before* headache cessation and were persisting *after* it, and symptoms whose onset was *after* headache cessation. We referred to the former as persistent symptoms and to the latter as true postdromes. Ninety-one per cent of patients reported persistent symptoms, with a mean of 6.0 and a median of 2, asthenia, pallor, cognitive difficulties, anorexia, somnolence, and nausea being the more frequently reported. True postdromes were reported by 82% of patients, with a mean of 2.6 and a median of 2, thirst, somnolence, visual disturbances, food craving, paraesthesias, and ocular pain being the most frequent reported. Interestingly, several prodromal and postdromal symptoms are also encountered during the aura classic and/or accompany the headache phase. Functional imaging in migraine has showed that the activations in areas such as hypothalamus or brainstem may begin before headache onset and/or persist after headache relief. Thus, one may wonder whether prodromal and postdromal symptoms may indicate the involvement of the limbic system, dopaminergic pathways, the hypothalamus and the brainstem. Differences between children, adolescents and adults might contribute to the understanding of migraine neurobiology.

## Introduction

Migraine is one of the most debilitating medical conditions, both in adult and pediatric populations (1). In the former it has familial, societal, and work consequences, while it may impede leisure and scholar activities in the latter, with the specific and supplementary issue of school absenteeism (2, 3).

Phenotypical expressions of migraine vary greatly both in the adult and pediatric age range. In both populations, and probably more so in the latter, that migraine headaches are frequently associated with non-headache symptoms has been known for a long time (4). These are ultimately epitomized by episodic syndromes which may be associated with migraine (5). It is meaningful that the historical recognition of these episodic syndromes occurred far earlier in children than in adults.

Symptoms other than headache may occur during the four phases of the attack: the prodrome, the aura, the headache phase, and the postdrome (6). Even in adults, prodrome, and postdrome seem to have been neglected (7, 8). Even fewer studies have been dedicated to them in children and adolescents (7, 8).

This is probably unfortunate as they may provide an insight on migraine pathophysiology, particularly if one takes advantage of the developmental differences in both populations (6, 9). Here we propose to review the available data on the subject, in children, adolescents, and adults as well. We will strive to decipher the possible mechanisms underlying these symptoms and to do so from a developmental perspective.

## Methodological issues

Reviewing the available data on the prodrome (PS) and the postdrome (PD) in the pediatric and adult range is fraught with several difficulties, notably methodological, which can be enumerated as follows (10):

1. There are few studies available on the subject:

To our knowledge, only three studies dedicated to the PD in the pediatric population (two included children and adolescents only whereas the third concerned both adults and children (11–13)) are available. Data are even scarcer for PD, as there is only one pediatric study dedicated to them (14).

Even in adults, few studies on the subject have been conducted.

2. Most of these studies are fraught with biases due to methodological differences.

- Definitions for both PS and PD vary from one study to another,
- There are large differences in the populations studied:
  ◦ Children and adolescents vs. both adults and children/adolescents,
  ◦ General population vs. clinic based,
  ◦ Preselected vs. non-preselected patients,
  ◦ Variable sample size,
  ◦ Variable sex ratio,
- Retrospective vs. prospective study,
- Pre-established questionnaire vs. open responses.

The way of collecting data is a major issue. As retrospective data collection leads to recall bias, prospective studies using electronic diary would be more appropriate but difficult to carry out in children and adolescents. Data collection from retrospective studies may also lead to underestimate the actual prevalence of PS or PD in the sample. Questionnaires with a pre-established list are associated with a risk that some patients may discard some symptoms whereas open questionnaires may be fraught with patients being unable to regard some non-specific or poorly specific symptoms as PS or PD symptoms, or mistaking PS for triggers. The issue of cranial autonomic symptoms such as face changes (pallor, flushing, or dark rings under the eyes) is particularly tricky.

Furthermore, some of the difficulties may be heightened in children and adolescents in whom the characterization of such symptoms in children faces difficulties, notably when it comes to history taking, age-related differences in communication, and cognition.

To put it bluntly, there is little comparability between studies… but, in the same time, they contain interesting material that may bring fruitful answers to the issue.

## What have we learned from these studies?

Generally speaking, PS refers to symptoms preceding the onset of migraine headache whereas PD corresponds to symptoms which begin after headache cessation. PS symptoms are subjective symptoms which develop slowly. They can be categorized as cognitive, behavioral, or physical factors. They characterize the pre-ictal state and should not be confused with the migraine aura, nor with triggers as food craving, for example, may be mistaken as food triggering a headache. Many triggers reported by migraineurs (e.g., sleep deprivation, hunger, or bright light), may in fact represent PS of an already ongoing attack.

The ICHD-3 states that “Prodromal symptoms may begin hours or a day or two before the other symptoms of a migraine attack with aura. They include various combinations of fatigue, difficulty in concentrating, neck stiffness, sensitivity to light and/or sound, nausea, blurred vision, yawning, and pallor. The term “prodrome,” which has replaced “prodrome phase” or “prodromal symptoms,” does not include aura” and, later, “Postdromal symptoms, most commonly feeling tired or weary, difficulty with concentration and neck stiffness, may follow resolution of the headache, persisting for up to 48 h” (ICHD-3, p. 21) (4). Further in the glossary, one can read: Prodrome- “A symptomatic phase, lasting up to 48 h, occurring before the onset of pain in migraine without aura or before the aura in migraine with aura. Among the common prodromal symptoms are fatigue, elated or depressed mood, unusual hunger, and cravings for certain foods” (ICHD-3, p. 211) (4). Whereas, the postdrome was not even defined in the glossary of terms in ICHD-3 beta, it appeared in the ICHD-3 glossary which states: Postdrome: “A symptomatic phase, lasting up to 48 h, following the resolution of pain in migraine attacks with or without aura. Among the common postdromal symptoms are feeling tired or weary, difficulty with concentration and neck stiffness” (ICHD-3, p. 210) (4, 15).

### The Migraine Prodrome

#### Pediatric Studies

We searched for the prevalence of 15 prodromal symptoms using a telephone questionnaire in 103 children and adolescents (< 18 years) suffering from migraine (with (MA) and/or without aura (MO), but not chronic migraine) according to the ICHD-II criteria, randomly drawn from a clinic-based database. Each interview concerned the migraine patient and one of his/her parents. The definition of prodrome was the same as in the ICHD-3 glossary (see above). The questionnaire comprised two parts; part 1 addressed migraine characteristics, part 2 listed 15 possible prodromal symptoms selected from the pediatric and adult literature. Patients were educated to distinguish prodromal from aura symptoms. Patients had to answer five questions pertaining to each prodromal symptom reported [see (11) for details and statistical methods]. Written informed consent was waived, as per national guidelines at the time of data collection.

**Figure 1:**
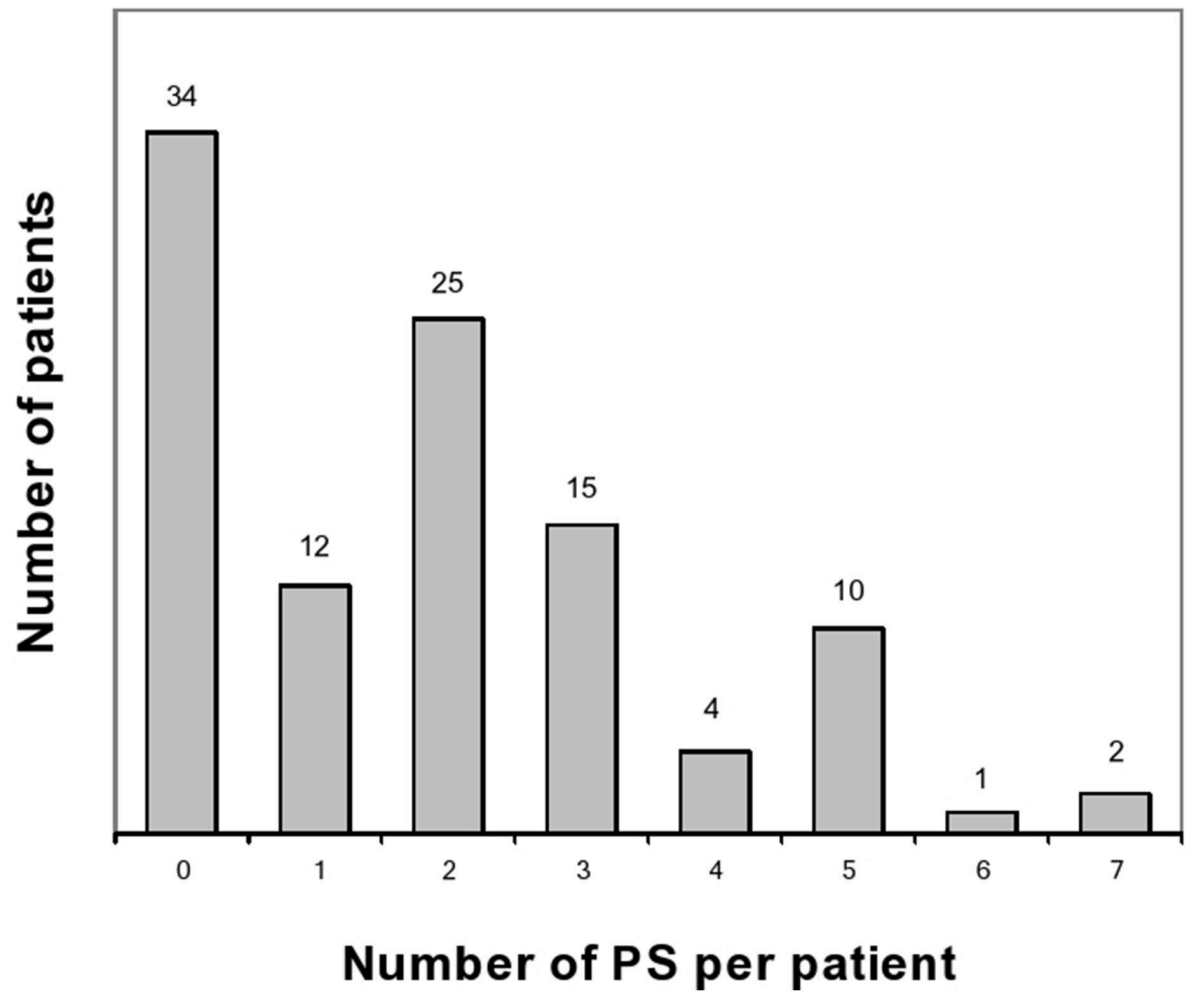
Number of prodromal symptoms per patient. PS, prodromal symptoms.

**Figure 2:**
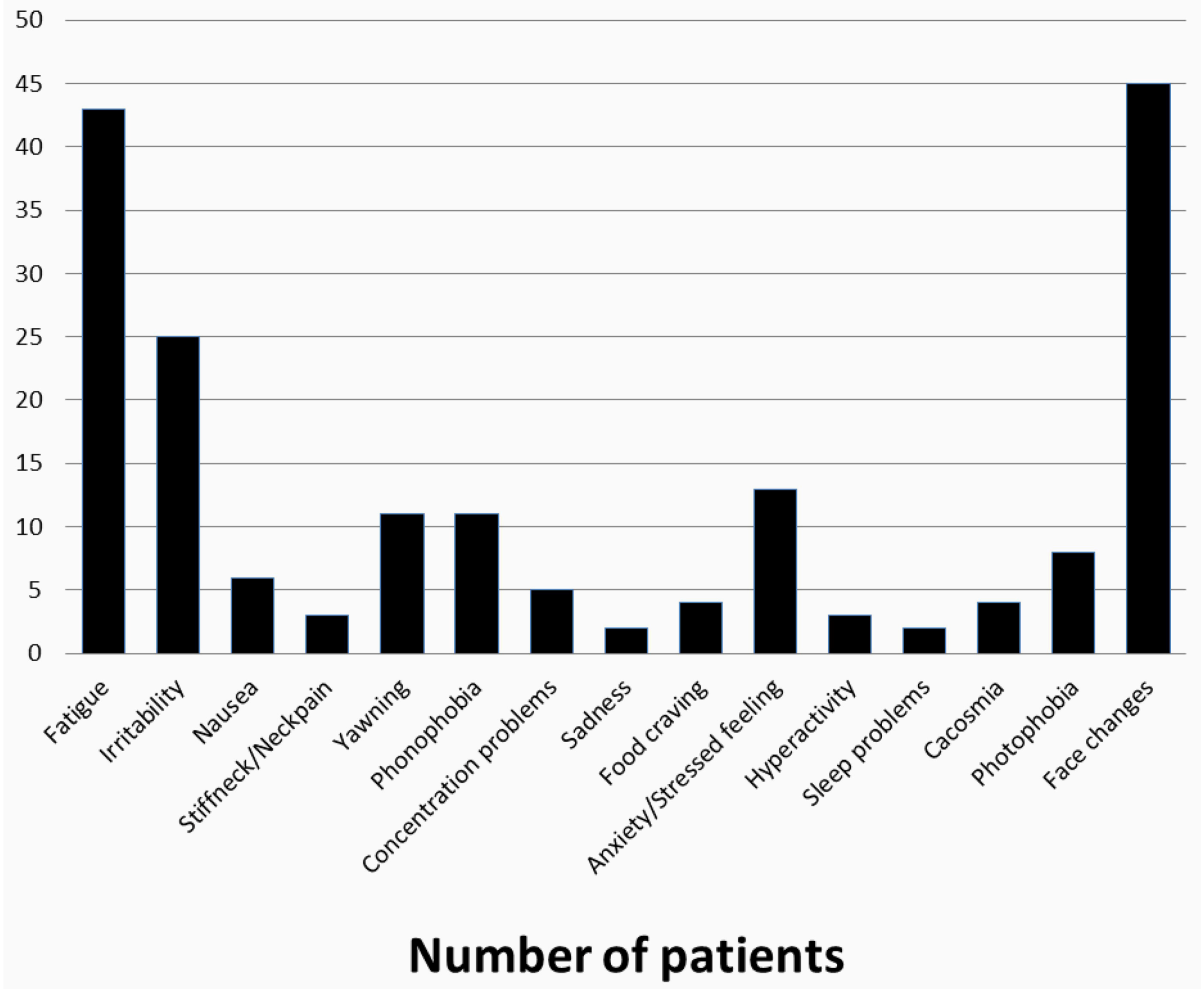
Prevalence of prodromal symptoms.

These results have been published elsewhere (11). In short, we included 103 patients. Table 1 shows main results. Prodrome consisted of one or more, and two or more prodromal symptoms for, respectively, 69 (67%) and 57 (55%) patients (Figures 1, 2). As for frequency, using the following scale: rarely, often, very often, and always when prodromal symptoms occurred, respectively in >0- < 1/3 attacks, 1/3–2/3, >2/3- < 1 or in each attack, the corresponding distribution was, respectively: 15, 11, 10, and 64% of the 69 subjects who had prodrome. There was no statistically significant link with gender, migraine subtype and mean monthly attack frequency. As for gender, 72% of boys and 65% of girls had prodrome.

**Table 1:** Migraine and demographic properties of patients reporting at least one prodromal symptom (*n* = 103).

	**Subgroups**	***N* (%)**	**Number of individuals with at least one prodromal symptom (%)**	**OR [CI]**	***p*-value**
Total population		103	69	
Sex	Male	57 (55%)	40 (70%)	
	Female	46 (45%)	29 (63%)	1.4 [0.6–3.1]	0.6
Age (years)	< 6	3 (3%) 1 (33%)		
	6–12	41 (40%)	26 (63%)	
	>12	59 (57%)	42 (71%)	1.6 [0.7–3.6]	0.4
Number of migraine attacks per month	< 1	29 (28%)	23 (79%)	
	1–2	26 (25%)	15 (58%)	
	3–4	22 (21%)	12 (55%)	
	5–6	13 (13%)	9 (69%)	
	7–8	9 (9%) 7 (77%)		
	9-10	4 (4%) 3 (75%)		
	>10	0 (0%) 0 (0%)		1.5 [0.5–3.9]	0.6
Migraine subtype	MO	11 (11%)	8 (73%)	
	MA	69 (67%)	45 (65%)	
	MO and MA	23 (22%)	16 (70%)	1.4 [0.3–5.9]	0.9

*N, number of patients. OR, odd ratio; CI, confidence interval*.

The main difference in design in the Karsan study (12), as compared to the other two studies (11, 13) was that patients were preselected as having prodrome by reviewing clinic letters from the initial consultation. The authors argued they wanted to develop “a better understanding of the range of symptoms when they were present.” Moreover, included patients (children and adolescents) not only suffered from migraine but also from New Daily Persistent Headache “with migrainous features.” Exclusion criteria included typical migraine aura and cranial autonomic symptoms. In this study, prodromal symptoms were defined “as symptoms recognized as occurring prior to the onset of pain and any non-migraine defining features occurring during the pain” with the exclusion of “any cranial autonomic features because of their discrete pathophysiology” (12). Of note, chronic migraine and episodic migraine accounted for 58 and 29% of the diagnoses, respectively. New Daily Persistent Headache with migrainous features (8%) and hemiplegic migraine (5%). Thirty one percent of patients reported a history of infantile colic, which accounted for the most frequent childhood episodic syndrome associated with migraine. The commonest number of reported prodromal symptoms was two. Two or more prodromal symptoms were reported by 85% of patients. Four prodromal symptoms were reported by >30% of patients: fatigue, mood change, yawning, and concentration difficulty.

Both children and adults were included (mean age 45 ± 17 years [5–96]) in a third study (13). The sample study consisted of patients suffering from migraine seen by neurologists at outpatient headache clinics in six Finnish cities with at least three first-degree relatives with possible migraine. The questionnaire comprised 14 predefined prodromal symptoms. The pediatric sample represented 6.2% (137/2219) of the total sample. Seventy-one of children and adolescents had had at least one prodrome symptom vs. 77% for the whole sample (mean 3.0 ± 2.9). Unfortunately, data pertaining to the pediatric sample were not further detailed by the authors in the article. Considering the whole sample (children and adults), prodromal symptoms were more than twice more frequent in migraine vs. non-migraine patients. Patients with MA had more prodromal symptoms (79%) vs. those with MO (75%; mean, respectively 3.3 vs. 2.7). Interestingly the subgroup with the lesser rate of prodromal symptoms was the typical aura without headache patients (frequency: 41%, mean number of prodromal symptoms: 0.8) whereas patients suffering from hemiplegic migraine had the greater rate of prodromal symptoms (frequency: 93%, mean number of prodromal symptoms: 5; *p* < 0.001). PS was more frequent in females. Females had also a higher number of prodromal symptoms unlike other studies, a tendency that may have been revealed by the large size of the sample. There was an inverse tendency for scintillating scotoma. A limit to the study was that the sample was skewed toward larger hereditary burden and more severe migraine. Moreover, face changes were not included in the list of predefined prodromal symptoms.

#### Comparison to Adult Studies

The proportion of pediatric migraine patients reporting PS was available in two studies only and was comparable: 67% (11) and 71% (13). In the Celeste study which included 398 children and adolescents suffering from primary headache (78.5% with migraine or probable migraine), only 11.8% of patients reported PS (11). These data are difficult to compare to adult studies, in which prevalence vary from 9 to 88% (Table 2) (16, 18, 19, 21–24, 26, 27). In population-based adult studies rates range from 12% in patients with MO to 18% in patients with MA (23). The mean number of prodromal symptoms reported per patient varies between 1.8 and 3. A lower figure was reported in the study by Schoonman et al: 3.2 (26). Whereas, PS occurs mainly in the 5–12 years age range, one may suppose that some of younger children may not be able to verbally express a symptom, as Mortimer et al. already noted (29). However, children as young as 18 month have been able to report PS (12). Interestingly, Laurell et al. found that PS rate was age-dependent and were able to estimate that the odds of experiencing PS increased by 1.0% per year (13). Conversely, a source of underdiagnosis (or misdiagnosis) lies in the fact that parents may not regard some symptoms as prodromal symptoms. It is worth underlying that only an external observer could identify some prodromal symptoms reported by the child (such as face changes in our study), rendering this finding notably dependent from the study design.

**Table 2:** Studies of prodromal symptoms.

**References**	**Type of study**	**P/R**	***N***	**Sample**	**PS symptoms**	**Comments/other results**
Cuvellier et al. (11)	Telephone Questionnaire/Checklist	R	103	Clinical sample of Children/adolescent with migraine	Face changes (44%), fatigue (42%), irritability (24%)	Frequency of PS trended higher with age but not statistically significant (*p* = 0.4). Differences by gender and migrainesubtype not statistically significant PS reported by 75% aged 12 and older, 68% in 6–12 age range, 33% in those < 6.
Karsan et al. (12)	Clinical letter	R	100	Clinical sample with migraine (episodic/chronic, NDPH)	Fatigue (62%), mood change (55%), neck stiffness (33%), and yawning (30%)	Preselected as having PS Infants as young as 18 months reported PS.
Amery et al. (16)	Unstructured recall and checklist	R	149	Population-based sample with migraine	PS–50% of patients with following PS the day before attack: adynamia, pallor, photophobia, phonophobia, hyperesthesia, shivers, taciturn, inactive, intolerant, intellectual disturbance	
Blau (17)	Oral questioning	R	50	Clinical sample	Yawning, tiredness, mood change	Prevalence:34%
Drummond and Lance (18)	Oral questioning	R	530	Clinical sample	Mood change, appetite change, changes of alertness	Prevalence: 30%
Giffin et al. (19)	Electronic diary	P	97	Clinical sample	Tiredness, concentration difficulties, stiff neck, light sensitivity, sound sensitivity	Preselected as having PS
Houtveen and Sorbi (20)	Electronic diary	P	93	Clinical sample	Increase in sensory sensitivity, pain/stiffness, fatigue, negative affect in the 12 h prior to attack	
Kelman (21)	Interview	R	893	Clinical sample with migraine	Fatigue (25.6%); mood change (23.4%); head pain, aching, twitching (5.6%)	No gender difference in frequency
Quintela et al. (22)	Questionnaire	R	100	GP surgery	Anxiety, phonophobia, irritability, low mood, yawning	Prevalence: 84%
Rasmussen and Olesen (23)	Interview & Questionnaire	R	1,000	Population	Low spirit, tiredness, increased activity, depression	Prevalence: 14%
Russell et al. (24)	Face-to-face/telephone interview	R	484	Clinical sample	Increased activity, low spirit, tiredness, depression, particular eating habits, irritability, yawning	Prevalence: 9%
Santoro et al. (25)	Self-report	R	100	Clinical sample with migraine	PS	Thirty-three percent of patients affected by migraine without aura reported PS in at least 50% of attacks. This subset reported a higher average number of trigger factors relative to other patients
Schoonman et al. (26)	Questionnaire	R	461	Clinical sample	Fatigue, phonophobia, yawning	Prevalence: 87%
Waelkens (27)	Questionnaire	P	49	Clinical sample	Irritability, depression, fatigue, hunger, bulimia, yawning	Prevalence: 88%
Wöber et al. (28)	Paper diary	P	327	Population	Muscle tension in the neck, stress, tension, fatigue	

*P, prospective; R, retrospective; N, number of patients; NDPH, New Daily Persistent Headache; GP, General Practitioner*.

In our study we found that face changes were the more frequent prodromal symptoms (44%) reported. Face changes (pallor, shadows under the eyes) seem to be peculiar to children and adolescents, as they have rarely been reported as prodromal symptoms by adults (19, 30). One may suppose that parents are more attentive to their child appearance due to legitimate concern whereas adults in the midst of an attack are not prone to look at themselves in a mirror. This is indeed a study bias easily missed between self-reporting and reporting by a third party. Inter study comparison precludes further definite conclusions; e.g., Karsan et al. excluded *a priori* cranial autonomic features from their study, pointing out that face changes may be cranial autonomic features, a statement we fully agree with (12).

In the Celeste study, the commonest prodromal symptoms were a feeling of great tiredness, irritability, yawning or sighing, balance disturbance, and mood change (31). The other most frequently reported prodromal symptoms were fatigue [62% (12), 42% (11), mood change (55% (12)], neck stiffness [33% (12)], and irritability [24% (11), 10% (12)]. Fatigue and irritability have been frequently reported in adult studies, with rates of 72% (19), 46.5% (21), and 25.6% (25) for fatigue, and 23.4% (25) for irritability. By contrast, some prodromal symptoms which were reported in adults, such as behavior changes, phonophobia, and gastrointestinal symptoms, were rarely reported in pediatric subjects. One may wonder if these findings represent an age-dependent feature or stem from methodological differences between studies.

As regards the constancy of the association of PS the constancy of PS being associated with the migraine attack, it concerned 64% of patients in our study, a figure higher than those reported in adults. In another study conducted in an outpatient clinic (*n* = 460 adult migraine patients), PS preceded migraine attacks in more than 2/3 of events in 46%; in this subgroup PS was followed by an attack in more than 2/3 of cases in 68% or more of the subjects, which was consistent with other findings reported in adults (26). This raises the issue of the predictability of the imminence of the attack (see below). Another issue is the consistency of PS phenomenology from an attack to the next one. To our knowledge this point has not been studied in pediatric samples but in adults, Quintela et al. showed that PS was reproducible across different migraine attacks (22), which allows self-prediction. Self-prediction is the ability by the migraine patient to assess the probability that he/she will have an attack over a defined time period. It may rely on triggers, PS features, or other considerations. To our knowledge, the question of self-prediction has not been studied in pediatric population.

The prevalence of prodromal symptoms did not differ with gender (11, 12), in contrast with the studies by Schoonman et al. (26) and Laurell et al. (13), where females reported more prodromal symptoms than males. Perhaps, the sex ratio may account for this difference, as there was a majority of women in adult studies, whereas boys were predominant in our study.

### The Migraine Postdrome

#### Pediatric Studies

**Figure 3:**
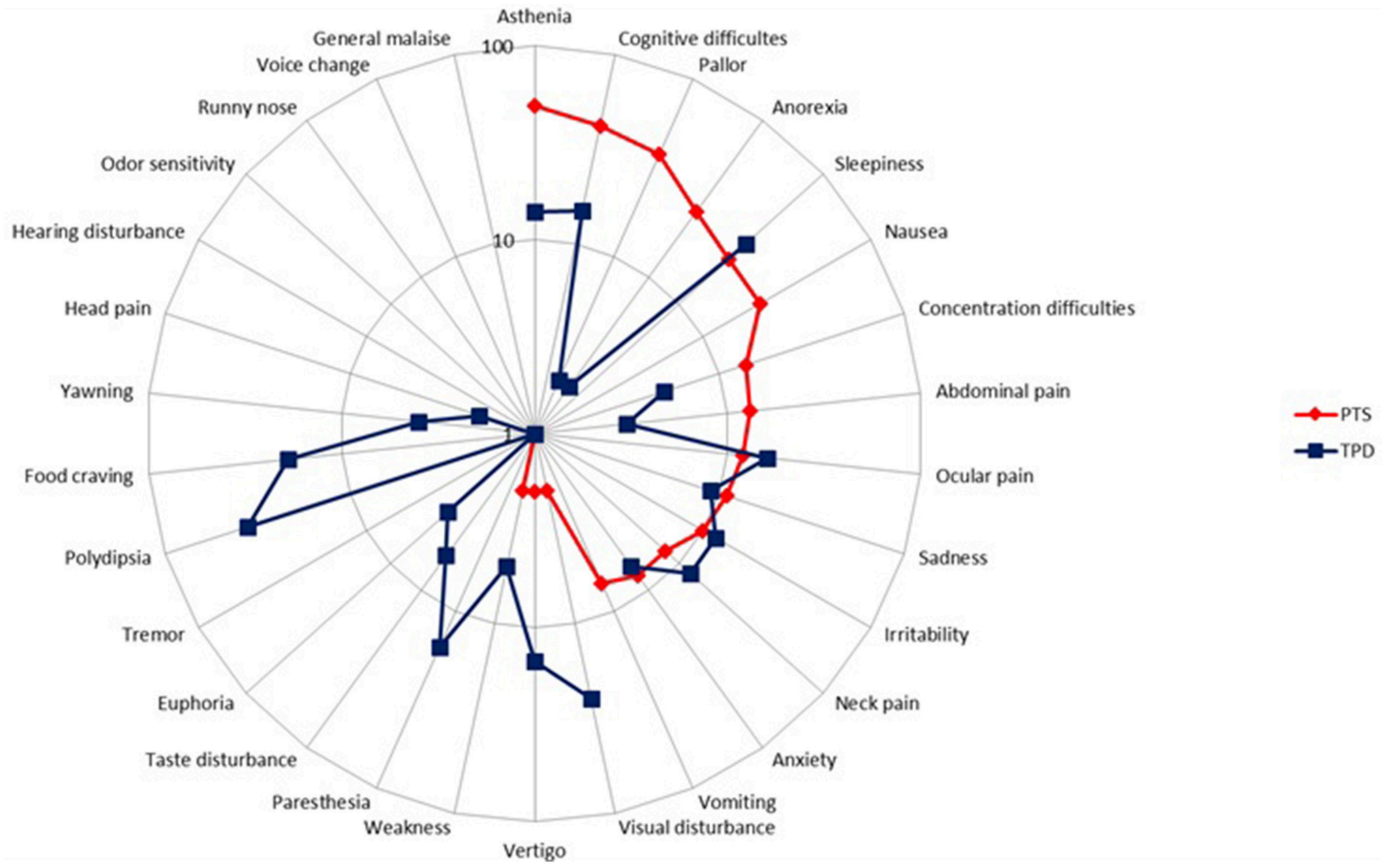
Frequency of persistent symptoms and true postdromes among pediatric migraineurs (*n* = 100). PTS, persistent symptoms; TPD, true postdromes.

Our preliminary research on the PD showed that children and adolescents reported two groups of symptoms occurring during the resolution phase of the migraine headache: symptoms that had begun before and went on after migraine headache had subsidized, and symptoms that began strictly after headache cessation. Thus, we decided to embark on their study and instructed both patients and parents to distinguish separately both sets of symptoms, referring to the formers as persistent symptoms (PTS) and to the latters as true postdromal symptoms (TPD). Methods were similar to our study on prodrome [to be included patients, who were randomly selected from my database of headache patients (8), had to be < 18 years, to fulfill the ICHD-3 beta criteria for pediatric migraine without typical aura (MO) and/or with aura (MA) at the time of study (i.e., ICHD-3 beta 1.1 and/or 1.2.1), but not chronic migraine, and not be on preventive drugs for migraine or any other medication]. Patients and/or their parents were first informed of the study objective and design. We reviewed with both the phases of migraine, including the concept of PD. We particularly instructed both patient and parents to distinguish separately PTS and TPD. The questionnaire comprised two parts; part 1 addressed migraine characteristics, part 2 listed 31 resolution phase symptoms selected from the adult literature. This list included behavioral, dietary, environmental, infectious, traumatic, hormonal factors, and other symptoms. Patients had to answer five questions pertaining to each postdromal symptom reported (Supplementary Material). All patients and their parent(s) provided informed written consent for participation in the study, which was approved by the Ethics Committee of Lille Faculty of Medicine. The results have been published elsewhere [see (14) for details and statistical methods] and we will briefly summarized them. Included patients consisted of 100 children and adolescents (49 boys), with an age range of 4–17 and a mean age of 10.5 years. Migraine subtype distribution (MO, MA, both MO and MA) was, respectively: 66 (66%), 26 (26%), and 8 (8%) patients. Thirty-three (33%), 50 (50%), 7 (7%), 5 (5%), and 5 (5%) patients had a monthly number of attacks of, respectively: < 2, 2- < 4, 5- < 7, 7- < 9, and 9- < 15. The interviewed parent was mainly the mother (95%). Most patients had either PTS (*N* = 80) or TPD (*N* = 82). Asthenia, cognitive difficulties, pallor, cognitive slowing, anorexia, sleepiness, and nausea were the most frequently cited PTS, by 49, 42, 38, 28, 26, 22, and 22% of patients, respectively (Figure 3). The median number of PTS differed according to migraine subtype distribution: 2 [0–10], 3 [0–8], and 3 [0–5] in patients with MO, MA, and both MO and MA, respectively (*p* = 0.60). Thirst, sleepiness, visual disturbances, food craving, paresthesias, and ocular pain were the most frequently cited TPD, by 36, 36, 25, 19, 16, and 16% of patients, respectively (Figure 3). The median number of TPD differed significantly according to migraine subtype distribution: 2 [0–11], 3 [0–9], and 1 [0–3] in patients with MO, MA, and both MO and MA, respectively (*p* = 0.03). Onset of TPD occurred < 30 min after migraine headache cessation in 95% of patients. Table 3 presents time data available in the 82 patients/parents capable to specify TPD duration, accounting for 257 TPD.

**Table 3:** Frequency and duration of persistent symptoms/true postdromes.

		**PTS (*n*, %)**	**TPD (*n*, %)**
**DURATION OF PERSISTENT SYMPTOMS/TRUE POSTDROMES**			
1	<3 h	100 (34.4%)	185 (72.0%)
2	3 to < 6 h	55 (18.9%)	28 (10.9%)
3	6 to < 12 h	52 (17.9%)	28 (10.9%)
4	12 to < 24 h	83 (28.5%)	11 (4.3%)
5	≥24 h	1 (0.3%)	5 (1.9%)
		291 (100%)	257 (100%)
**FREQUENCY OF PERSISTENT SYMPTOMS/POSTDROMES AS A FUNCTION OF MIGRAINE ATTACKS**			
1	Always	2 (0.7%)	2 (0.8%)
2	Very often	19 (6.5%)	52 (20.2%)
3	Often	128 (44.0%)	125 (48.6%)
4	Rarely	142 (48.8%)	78 (30.4%)
		291 (100%)	257 (100%)

*PTS, Persistent symptoms*.

*TPD, True postdromal symptoms*.

Several results reach statistical significance: PTS were reported more frequently by boys than girls (94 vs. 67%, *p* < 0.001), mean number of PTS was greater in boys (mean: 3.0 vs. 2.0 for girls, *p* = 0.003). Pooled PTS+TPD lasted less in girls (*p* < 0.001).

Grouping together symptoms which are established prodromal symptoms (sadness, neck pain, food craving, concentration difficulties, asthenia, yawning), aura symptoms (visual disturbances, paresthesias), and classic accompanying symptoms of the headache phase (pallor, nausea, vomiting, abdominal pain, anorexia, irritability, dizziness), we found that they were reported as PTS in 82, 3, and 118 cases and as TPD in 63, 41, and 34 cases, respectively (*p* < 0.001) (Table 4).

**Table 4:** Frequency of persistent symptoms/true postdromes as a function of headache phase category.

	**Persistent symptoms**	**True postdromes**
Premonitory symptoms^a^	82	63
Aura symptoms^b^	3	41
Accompanying signs^c^	118	34

a *Concentration problems, food craving, sadness, stiff neck/neck pain, yawning, asthenia*.

b *Visual symptoms, paresthesias*.

c *Nausea, pallor, vomiting, abdominal pain, anorexia, irritability, dizziness. (p < 0.0001)*.

Retrospective character, small sample size, and tertiary unit recruitment were the main limits of our study. One may also underline that some aspects of time and memory were not perfectly handled in the pediatric age group.

#### Comparison to Adult Studies

From our results we could conclude that children and adolescents with migraine frequently experienced both PD symptoms subtypes. It is also of note that the child falls asleep in as many as 60% of children, which aborts the migraine attack and thus avoids or masks the PD (32). In the absence of another pediatric study, we can but compare our results with adult studies (Table 5). However, this is hampered by several difficulties; first, we are unaware of an adult study using the distinction we made between PTS and TPD. Second, PD definition is variable between studies. With these reserves in mind, most adult patients had PD: respectively 94, 68, 80, and 81% in the studies by Blau (33), Kelman (34), Giffin et al. (35), and Quintela et al. (22). The latter was a prospective daily electronic diary study, where the PD was defined as “the time between headache resolution and feeling completely back to normal” (35).

**Table 5:** Postdrome adult studies.

**References**	**Type of study**	**P/R**	***N***	**Sample**	**Postdrome symptoms**	**Duration**	**Prevalence**
Blau (33)	Interview	R	50	Clinical sample	Mood variations, muscular weakness, abnormal appetite, yawning, tiredness, and changes in fluid balance.	1 h−4 d	94%
Giffin (19)	Electronic diary study	P	120	Clinical sample	Tiredness or weariness (88%), difficulty with concentration (56%) and stiff neck (42%).	≥24 h in 93%	81%
Kelman (34)	Structured interview	R	827	Clinical sample	Tiredness (71.8%), head pain (33.1%), cognitive difficulties (11.7%), “hangover” (10.7%)	56% < 12 h, 32% 12 ≤ 24 h, 12% > 24 h	68%
Quintela (22)	Interview	R	100	GP clinical sample	Asthenia (55%), tiredness (46%), somnolence (29%), concentration difficulties (28%)		80%
Giffin (35)	Electronic diary study	P	120	Clinical sample	Tiredness or weariness (88%), difficulty with concentration (56%), and stiff neck (42%).	≥24 h in 93%	85%

*P, prospective; R, retrospective; N, number of patients; GP, general practitioner*.

PD duration was longer in adults, with a mean of 18 h (Blau) and 25.2 h (Kelman) (33, 34). Duration of both PTS and TPD was < 12 h in most patients (14). In one small study (*n* = 34), the PD lasted between 30 min and 6 h for most symptoms, but some patients could experience PD which lasted up to 4 days for (33). Results were similar in a recent electronic diary study, with 54% of patients having a PD duration < 6 h whereas PD duration was >24 h in only 7% of patients (35). TPD phenomenology was notably different from that reported in adult PD. The most commonly PD symptoms reported by adult patients are tiredness, concentration difficulty, and neck stiffness (8). Asthenia, somnolence, phonophobia, photophobia, unhappiness, and yawning (22), head pain, cognitive difficulties, “hangover,” gastrointestinal symptoms, mood change, and weakness (34), nausea, physical weakness (36), tiredness (22, 34, 35), concentration difficulties (34, 36) have also been reported. In our study PTS were more frequent in patients with MA only compared to MO only and both MO and MA, as in some adult studies (22, 34).

## So What?

Children, adolescents, and adults suffering from migraine do have PS and PD frequently. Bearing in mind the great heterogeneity between studies, prodromal symptoms are roughly the same in the three age ranges, with the notable exception of face changes which seem to be a pediatric peculiarity, but so far they have been reported in our study only. As to PD, it is difficult to draw definite conclusions with only one study but let us notice that whereas temporal characteristics of PTS/TPD shared some similarities, with the obvious exception of time lag, as expected due to the definitions employed, the nature of TPD and PTS showed differences, as shown in Table 3.

Some authors of adult studies have attempted to group PS and PD symptoms according to general categories such as cognitive or sleep-related, migraine-like and sensory sensitivities, and other homeostatic symptoms. The same approach can be made in the pediatric population.

It is remarkable that some PS and PD symptoms share similarities, if not identities. Karsan and Goadsby have proposed to group PS starting simultaneously with pain, or occurring during the pain itself under the umbrella term “premonitory-like” as they “have observed that they can start simultaneously with pain, or occur during the pain itself” (37). Some PTS and TPD reported in our study dedicated to the PD in children and adolescents are clearly reminiscent of PS symptoms (14). Several adult studies dedicated to the PD of a migraine attack led to comparable conclusions (34, 35, 38). This suggests that PS and PD may have pathophysiological similarities and be generated by the activation of shared neural networks.

### Possible Window on Migraine Pathophysiology and Developmental Differences

#### Neuronal Networks Into Play

Understanding the factors associated with headache beginning and cessation might provide insights into the mechanisms of attack initiation and termination, and perhaps shed light on the issue of why there being different subtypes of migraine (39–41). The hypothesis of (a) possible migraine generator(s) has gained credit over the last years and one may raise the issue whether PS reflect the early activation of them while PD in the same way would indicate that some of these networks would still be ongoing once the headache has ceased. The chronology of this process may indeed prove more interesting; in other words, does the sequence progression of PS and PD reflect the successive activation of generators? And what degree of dependency do they share? Once activated, are they able to withdraw from their counterparts and to which degree?

The brainstem seems a good candidate in the generation of some prodromal symptoms, such as yawning, mood changes, irritability, hyperactivity, and sleep disturbances. Other prodromal symptoms point to the hypothalamus (thirst, food craving, sleep disturbances, pissing, and neck stiffness). Some of the former prodromal symptoms reflect dopaminergic hypersensitivity and are mediated by nitric oxide pathways (42). Del Zompo et al. have shown that alterations in dopaminergic neurotransmission can modulate clinical susceptibility to migraine, at least in some migraine patients, and dopamine can play a key role in activating the biochemical cascade leading to the PS, and ultimately in the migraine attack (43). Vasopressin and the orexins are alternative candidates, through their connections with the limbic system. Some authors (30) hypothesized that many prodromal symptoms might share a common biological basis related to the headache phase (some brainstem nuclei, which regulate the amount of pain as well as other sensory inputs, may be disinhibited, thus disrupting their associated motor and autonomic activities). As a result, it would be necessary that a critical physiological threshold be reached to induce the full-blown migraine headache (19). Other prodromal symptoms (emotional change, fatigue, and concentration difficulties) may reflect the involvement of the limbic system, whereas other brainstem nuclei brain structures outside of pain pathways may, for instance, account for nausea. Furthermore, hypothalamic-brainstem connections may account for fatigue and sleep and wakefulness disturbance may also arise from hypothalamic-brainstem connections (44).

Blau saw in the PD the converse process of the PS. He additionally proposed that it might reflect a slow decline in migraine processes and that the diversity of PD symptoms could be accounted for by an involvement of the whole brain (38), notably the frontal lobes and the hypothalamus. Thus, the multitude of symptoms reported by patients in the PD could be explained by a diffuse cortical and subcortical involvement. Bose et al. have proposed that the PD might be explained by widespread vasoconstriction mediated by an alpha2-adrenoreceptor mechanism mediated by activation of brainstem nuclei. As one of the major neuromodulatory structures of the brainstem implicated in the regulation of cortical function and the modulation of responses to afferent traffic, the locus coeruleus might play a pivotal role in this process (8). An alternative hypothesis involves cortical spreading depression. As persistent hypoperfusion following cortical spreading depression has been demonstrated (45), Bose et al. proposed that this hypoperfusion shown during the migraine attack might be related to cortical spreading depression (8).

#### Functional Imaging Studies

Several functional imaging studies performed in adults have provided some support to the previous assertions. As regards PS, one study by Maniyar et al. using positron emission tomography, has showed that several brain areas were activated before headache. These included subcortical (posterior hypothalamus, ventral tegmental area, periaqueductal gray matter, dorsal pons, putamen, caudate nucleus, and the pulvinar nucleus of the thalamus) but also cortical areas (occipital cortex, frontal, prefrontal, temporal, parietal cortex, anterior cingulate, and posterior cingulate) (46). These findings outlined the early involvement of the hypothalamus and brainstem (especially dorsal rostral pons and periaqueductal gray matter) in the mediation of the migraine attack. The same team conducted a second positron emission tomography study which showed that patients who experienced nausea during the PS showed activation in rostral dorsal medulla and periaqueductal gray, which was absent in patients without nausea (47). Using a similar design, Maniyar et al. assigned the origin of photophobia to the visual cortex during the premonitory phase of migraine in the absence of headache (48). With a completely different design, investigating a single patient daily over a whole month, Schulte and May found hypothalamic activation within the 24 h before headache onset as compared with the interictal state (49).

Less functional imaging study has been dedicated to the PD. Using arterial spin labeling MRI, Bose et al. have shown that cerebral perfusion was diffusely reduced during postdrome (50). The authors concluded that their results might be explained by the participation of several brain areas, both in the cortex and the brainstem, namely “the superior frontal gyrus, medial frontal gyrus, middle frontal gyrus, putamen, superior temporal gyrus, middle temporal gyrus, inferior temporal gyrus, posterior cingulate, anterior cingulate, thalamus, hypothalamus, and midbrain” (8). Alternatively, other functional imaging studies have shown that the activations in areas such as hypothalamus (51) or brainstem may persist after headache relief by sumatriptan (52), lending support to the hypothesis that some neural networks remain active when the headache has stopped.

For evident ethical grounds reasons, such studies are lacking in children and adolescents. This is all the more regrettable as the localization of prodromal and postdromal symptoms is harder to assess in a pediatric brain. It would be interesting to know whether adult findings can be transposable in children and adolescents, moreover in an age dependent fashion, in view of changes in brain development and maturation. Such studies would be invaluable in young children who are unable of identifying “subtle” symptoms due to their cognitive developmental level.

### Temporal Meltdown?

Perhaps, one may imagine that some prodromal symptoms start before headache onset, go on during the headache phase, more or less masked with relative success by the headache and accompanying symptoms, and reappear at the forefront after headache cessation under the mask of the PD. In short, everything would happen as if the classical temporal relationship between PS, aura and headache had been challenged. In this view, symptoms occurring during the PD (both PTS and/or TPD) would have made a “temporal mistake” and would not have followed the expected pattern. Examining the classic temporal relationship between aura and headache, Viana et al. have recently shown that aura occurred after resolution of the headache phase in 9% of their patients (53). In my experience, the upheaval of the classic sequential order is also very common in children and adolescents. One may suppose that just like aura may occur during the headache phase or follow it, the same might hold true for PD (19). Hence the question: are PTS/TPD an extension or a recurrence of the aura symptoms, which would eventually be masked by the headache phase, or are there similar mechanisms between aura and PTS/TPD? One may wonder whether hypothalamic activation may occur in phases, reflecting upset temporal patterns of symptoms. Similar mechanisms might be at work for prodromal symptoms, possibly through connections between the trigeminovascular system and the midbrain and the amygdala. This would also explain why patients are more prone to have PS if they suffer from MA.

### A Developmental Explanation Attempt

Goadsby stated that “migraine is a disorder for life, from the more unsettled child with colic, to the late-life migrainous accompaniments” (54). And below: “Perhaps this explains why the disease has the same flavor all through life but runs at different temperatures” (54). Let us remember that PS symptoms have been reported in infants as young as 18 months (12).

One is tempted to link changes in migraine symptomatology with developmental features associated to brain maturation. In this perspective, how can we account for differences in PS and PD between adults, children and adolescents? First of all, it is more difficult to infer the pediatric cerebral localization of symptoms; all the more because to analyze symptoms phenomenology in children and adolescents may prove more difficult and because an age-dependent precise description is lacking so far. It is noteworthy that the well-known modification of migraine pain location, evolving from bilateral in children and adolescents to unilateral in adults, has not been explained so far (55). As Chakravarty et al. pointed: “It can only be postulated that this may be the result of differences in degree of brain maturation comprising myelination, new synapse formation and synaptic reorganization.” One may infer similar hypotheses explaining the pediatric peculiarities of PS and PD (55).

Data drawn from the study of a periodic syndrome such as abdominal migraine may be more in line with the issue under examination. Abdominal migraine is a childhood disorder which evolves to more usual migraine subtypes as the child gets older. Symptoms consist of abdominal pain, pallor, nausea, and vomiting, but usually not headache. Gastroparesis often accompanies attacks, the cause of which has not really been investigated, to our knowledge. Besides gastroparesis, other symptoms include abdominal pain, nausea, and vomiting. A dysfunction of the autonomic nervous system and the maturation of the autonomic nervous system with age may account for this transformation and the persistence of a core of similar symptoms. One could speculate that these changes might be explained by a modulatory influence on (a) common network(s), the latter changing with age due to maturational changes (and/or perhaps, due to targets changes). Triggering factors such as stress or excitement suggest the involvement of aminergic systems, such as locus coeruleus, in this process. In this way, migraine attacks would be initiated through dysautonomia. That the influence of sleep is more important in children and adolescents than in adults may be another hint. Sleep alterations constitute an important trigger of migraine attacks and many migraine attacks terminate with the child falling asleep and awakening pain-free (32). These data may be accounted for by corresponding changes in autonomic tone as the child ages. However, up to now, the longitudinal maturational evolution of the autonomic nervous system has not been determined (56).

Another candidate is the serotoninergic network. Serotonin plays a vital role as a neurotransmitter in adult brain. It appears earlier in development than other monoamine transmitter systems and its turnover rate is higher in the immature mammalian brain than at any other. It is also involved in the regulation of brain development, intervening in particular notably in the processes of long-term potentiation and synaptic plasticity. An additional issue is how neural circuits change during before and with puberty. Remembering that migraine often starts in adolescence, or attacks frequency is influenced by puberty, there is further need to investigate the potential effects of sex hormones (57). It should not be forgotten that the mechanisms underlying this activation of the three most important neuroendocrine axes involved in puberty (that is the hypothalamic-pituitary-gonadal axis, the hypothalamic-pituitary-adrenal axis, and the growth hormone-insulin like growth factor axis) are only partly understood. Complex interrelations between stimulatory (leptin, glutamate, serotonin, galanine, dopamine, norepinephrine) or inhibitory (neuropeptin Y, melatonin, GABA) factors are at play to control the timing of puberty onset. Among the modulator substances, adrenal hormones exert key roles in the regulation and trophicity of cell survival, differentiation, maturation, and synaptogenesis of the central nervous system (58).

It would be interesting to test these hypotheses with functional sequential and longitudinal imaging, but as previously said, this is actually unavailable. However, we dispose of both cross-sectional and longitudinal studies dedicated to event-related potentials. The measurement of event-related potentials to sensory stimuli (e.g., visual) and slow cortical potentials suggests altered maturation of cortical information processing (59, 60) in children with migraine.

Taking account of established comorbidities of migraine, such as attention deficit disorder, anxiety, depression, and immunological disorders may suggest supplementary hints. Attention enhancement with age reflects the increasing frontal influence of connectivity modifications in many brain regions. One of the most critical adjustments in adolescence is an increase in brain dopamine, particularly in the “reward” pathway that involves the ventral tegmental area, the nucleus accumbens, and connections through the limbic system and eventually the frontal cortex (61–63). Mood change may be associated with cingulate gyrus activation, perhaps with the involvement of some of its limbic connections (46, 64). Limbic structures mature more rapidly than prefrontal and frontal cortex (61). Of note in a developmental perspective is the role of the anterior cingulate cortex. Located in the frontal lobe which is known to mature belatedly in adolescence, it is involved in the emotional processing of pain. Development of frontal regions appears to occur more rapidly from early adolescence to middle adolescence (ages 12 to 17) than from childhood to early adolescence (ages 9 to 12). The prefrontal cortex contains neurons that influence the parasympathetic or sympathetic motor neurons; it also contains different neurons that project to diverse body compartments, suggesting links with the autonomic nervous system. Since the hypothalamus is connected in different ways to systems which modulate pain and also to the spinal trigeminal nuclei, the influence of these maturational changes may perhaps affect less the successive involvement of specific neural networks with aging, but, instead, the evolving changes in functional connectivity between neural networks as the child grows older which matters (65). Whereas, brain maturation may affect migraine symptoms phenomenology as time goes, conversely, migraine may influence the development of the brain.

Finally, it is noteworthy to note that several immunological changes have been identified to be altered or associated with migraine in children and adults (66), including increased levels of calcitonin gene-related peptide (67), decreased levels of coenzyme Q 10 (68), and hormonal changes (69–72). These may constitute fruitful ways of research.

Whereas, the underlying basis for “hyperexcitability” (better accounted for as a brain tendency to over-respond) in migraine is unclear, genetic factors are also at play. Several susceptibility gene variants have been identified. It is of interest that, among these genes, some may regulate synaptic development and plasticity, such as ASTN2 and FHL5 (73, 74).

## Possible implications

Understanding mechanisms and networks at play before attack onset may ultimately lead to new, more targeted and more efficacious therapeutic strategies. PS may constitute an ideal window for early treatment. Even in adults, data which support this statement are scarce but the efficacy in migraine prevention of naratriptan and dopamine antagonists is suggested by nonrandomized trials (75, 76). It should be interesting to undertake placebo-controlled, randomized trials to ascertain this hypothesis. Similarly, domperidone, a dopamine antagonist, may block a migraine attack, provided it is taken at least 6 h before the putative attack (77, 78). The fact that children and adolescents experience shorter migraine attacks as compared to adults makes this issue eminently sensitive. Developing new molecules which, given during the PS, could ultimately prevent pain onset, would represent a major breakthrough. However, to our knowledge, such studies are unavailable so far in children and adolescents.

It has been shown, in adult migraine sufferers, that nitroglycerin and pituitary adenylate-cyclase activating protein could induce postdromal symptoms, which are similar to those experienced during spontaneous attacks (42, 79). In the wake of the recent interest for pituitary adenylate-cyclase activating protein, researchers have designed molecules that target the PAC1 receptor. This may represent a new therapeutic avenue for migraine, as may also the understanding of neurobiological mechanisms that underlie PD.

## Conclusion

Clinicians should be alert to both PS and PD and learn to recognize them (and differentiate them from triggers) in order to better evaluate the whole burden of the migraine attack, but also reliably predict the impending onset of the attack. Similarly, one should educate parents to be attentive to and recognize early PS symptoms which are, for part, noticeable, which is all the more interesting in non-verbal patients such as young children. At the same time, new research seems necessary to better characterize both PS and PD symptoms with rigorous, prospective methods, ideally using electronic diary systems. This may allow a better estimation of the population prevalence of PS and PD in different age ranges. The reproducibility of these symptoms across serial attacks should also be studied as well as their probability at predicting an impending headache attack.

Finally, these studies should be more oriented in a developmental perspective. The answers to the following questions appear crucial: are there distinct PS and PD as a function of different age range (infancy, childhood, and adolescence vs. adults)? Are there distinct subgroups of patients which could be categorized according to their specific PS and/or PD phenomenology? How do these symptoms evolve with age? How are PS and PD related in these patients? Thinking at new ways to circumvent current hindrances in conducting functional brain imaging studies in the younger pediatric populations would certainly lead to further advances. Maybe the answer to these questions would help to decipher the complex interrelations between PS, aura, headache, and PD, and design new therapeutic strategies, in an age-dependent fashion, with the ultimate goal of reducing morbidity, negative impact on academic performance, and school absenteeism. This is all the more urgently needed in children where the therapeutic armentorium is reduced in comparison with adults. This is unbelievably an interesting and exciting area for future migraine research!

## Author Contributions

The author confirms being the sole contributor of this work and has approved it for publication.

### Conflict of Interest Statement

The author declares that the research was conducted in the absence of any commercial or financial relationships that could be construed as a potential conflict of interest.

## Abbreviations

MA: migraine with aura
MO: migraine without aura
PD: postdrome
PS: prodrome
PTS: persistent symptoms
TPD: true postdromes.
