# Supplementary material for: Pediatric vs. Adult Prodrome and Postdrome: A Window on Migraine Pathophysiology?
Source: Front Neurol. 2019 Mar 8;10:199. doi: 10.3389/fneur.2019.00199 (PMC6423905; doi:10.3389/fneur.2019.00199)
Supplement: Supplementary file 1 [file Data_Sheet_1.docx]

**Appendix**

**Questions 1**

*Which, among the following symptoms, are still present once the headache has subsided?*

**Questions 2***

*Can you specify, for each symptom, what is the time of onset?*

1. During migraine headache

2. <30 mn following migraine headache cessation

3. =<30 mn-<2 hours following migraine headache cessation

4. =<2 hours-<3 hours following migraine headache cessation

5. >=3 hours following migraine headache cessation

**Questions 3***

*Specify the duration of every symptom*

1. <3 hours

2. 3 to <6 hours

3. 6 to <12 hours

4. 12 to <24 hours

5. >=24 hours

**Questions 4***

*Specify the duration of every symptom, after migraine headache cessation*

1. <3 hours

2. 3 to <6 hours

3. 6 to <12 hours

4. 12 to <24 hours

5. >=24 hours

**Questions 5***

*Specify how often every symptom occurs during the migraine attack*

1. Always

2. Very often (>=2/3-<1 attacks)

3. Often (>=1/3-<2/3 attacks)

4. Rarely (>0-<1/3 attacks)

*****For each question, tick appropriate answer. Only one possible answer.
